# Novel flexible cap for application of transcranial electrical stimulation: a usability study

**DOI:** 10.1186/s12938-020-00792-1

**Published:** 2020-06-17

**Authors:** Alexander Hunold, Daniela Ortega, Klaus Schellhorn, Jens Haueisen

**Affiliations:** 1 Institute of Biomedical Engineering and Informatics, Technische Universität Ilmenau, 98693 Ilmenau, Germany; 2 Bioinstrumentation and Clinical Engineering Research Group, Universidad de Antioquia, Medellín, 050010 Colombia; 3 neuroConn GmbH, 98693 Ilmenau, Germany; 4 Hans Berger Department of Neurology, Biomagnetic Center, University Hospital Jena, 07747 Jena, Germany

**Keywords:** transcranial Electrical Stimulation, transcranial Direct Current Stimulation, Patient comfort, Reproducibility, Electrodes, Electric impedance

## Abstract

**Background:**

Advances in transcranial electrical stimulation (tES) are hampered by the conventional rubber electrodes manually attached to the head with rubber bands. This procedure limits montages to a few electrodes, is error prone with respect to electrode configurations and is burdensome for participants and operators. A newly developed flexible cap with integrated textile stimulation electrodes was compared to the conventional setup of rubber electrodes inserted into sponges fixated by rubber bands, with respect to usability and reliability. Two operators applied both setups to 20 healthy volunteers participating in the study. Electrode position and impedance measures as well as subjective evaluations from participants and operators were obtained throughout the stimulation sessions.

**Results:**

Our results demonstrated the superiority of the flexible cap by means of significantly higher electrode configuration reproducibility and a more efficient application. Both, operators and volunteers evaluated the flexible cap as easier to use and more comfortable to wear when compared to the conventional setup.

**Conclusion:**

In conclusion, the new cap improves existing and opens new application scenarios for tES.

## Background

Non-invasive techniques to modulate neuronal activity include the transcranial electrical (current) stimulation (tES) [1]. tES can be classified based on the current form to transcranial Alternating Current Stimulation (tACS), transcranial Direct Current Stimulation (tDCS), and transcranial Random Noise Stimulation (tRNS). The technique has applications to different neurological and psychiatric disorders such as stroke [2, 3], depression [4, 5], epilepsy [6, 7] and chronic pain [8, 9].

Transcranial electrical stimulation applies weak currents in the range of 1–2 mA via at least two electrodes to change the brain activity level. In early applications of tDCS, the anode was positioned above the motor cortex and cathode at a contralateral prefrontal position by rubber bands surrounding the head [10]. In order to establish a safe low impedance of the electrode–skin interface, the rubber electrodes were embedded in sponge pockets soaked with saline solution [11] or spread with conductive gel to contact the scalp.

These procedures, incorporating several pieces of equipment, are complex and require professionally trained personnel to perform them, even when applying fixation approaches based on caps [12] or headgears [13]. So far, these prerequisites prevent the transfer of tES to home-use scenarios. The cumbersome positioning of single electrodes generates errors in the electrodes’ placement and, in their effective areas, and it limits their applications to target different neuronal areas. In longitudinal studies, reproducibility in tES applications is relevant [14] and depends to a large extent on consistent positioning and placement. Generally, clinical studies are carried out by different operators, with different patients and in different places, thus the manual and unstable electrode placement has a negative effect on the reproducibility of the tests [15].

Moreover, the electrodes used in the conventional applications have a rectangular shape, which limited the ability to follow the curved surface of the scalp, resulting in partial displacement. Further, in new applications, the number of electrodes and their positions increase montage complexity, which is currently limited by the rubber bands [16].

Electrode applications with conductive gel [17] or adhesive layers [18] have been proposed. Electrode positioning has been suggested based on the international 10–20 system [19, 20]. However, these approaches limit degrees of freedom in electrode positioning due to the fixed layout of the electrodes [21]. Moreover, semi-rigid cap systems might not adapt to each head shape and size.

To overcome limitations in the electrodes configurations for tES applications, we propose a flexible cap with integrated textile stimulation electrodes, which can be produced in lot size one, and thus cap size and electrode positions can be customized to the need of each patient. Here, we compare the novel flexible cap (setup C) to the conventional rubber electrodes in sponge pockets fixed with rubber bands (setup R), with respect to usability and reliability.

## Results

On average, the placement took 3.9 min ± 3.4 min (mean ± std) with a 95% confidence interval of the mean value of [2.6; 5.2] min for setup C; it took 6.8 min ± 2.6 min with a 95% confidence interval of the mean value of [5.8; 7.8] min for setup R.

**Fig. 1:**
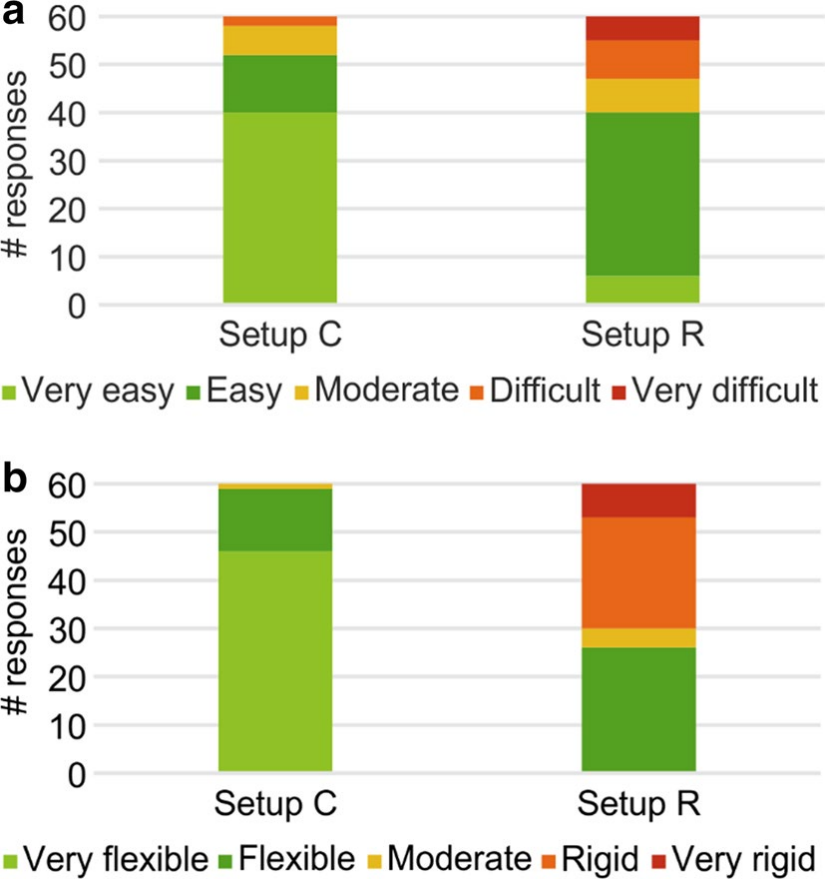
Histograms of operators’ responses on the evaluation for ease of application (**a**) and material flexibility (**b**). The total number of responses (*n* = 60) refers to the two operators conducting 30 sessions each (10 volunteers, each in three sessions)

Figure 1 shows the operators’ evaluations of the effort involved in application and the material flexibility in 60 tests. For ease of application (Fig. 1a), 87% (*n* = 52) of the responses described setup C as “very easy” or “easy”, and only 3% (*n* = 2) described it as “difficult” to fit the cap to the participant’s head, while setup R was considered “very easy” or “easy” in 66% (*n* = 40), “difficult” in 13.3% (*n* = 8), and “very difficult” in 8.3% (*n* = 5) of the responses. With respect to the material assessment, setup C was described as “very flexible” or “flexible” in 98% (*n* = 59) of the applications, and setup R was evaluated as “flexible” in 43.3% (*n* = 26) of the applications and as “very rigid” or “rigid” in 50% (*n* = 30) of the tests.

**Fig. 2:**
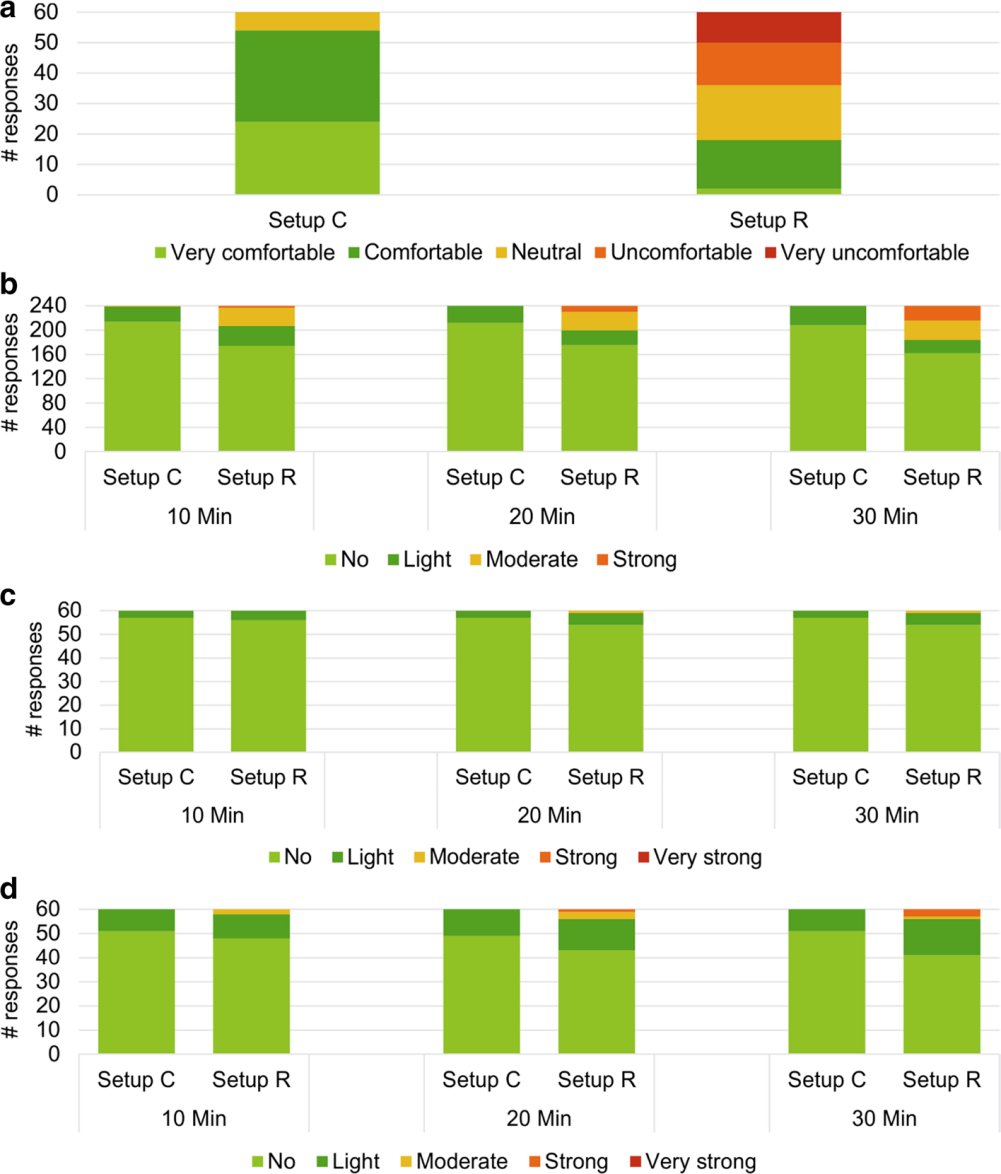
Histograms of participants' responses on the evaluation of comfort (*n* = 60) (**a**), pressure sensation at four considered locations (*n* = 240 from 60 recordings of 4 positions) (**b**), sweat (*n* = 60) (**c**), and itch (*n* = 60) (**d**) sensations

The participants evaluated the comfort of the two setups for each session, the pressure level in four positions (underneath the electrodes, at the temples, underneath the straps, other locations) and their perceptions of itching and sweating throughout the stimulation periods of 30 min. Figure 2a shows that 90% (*n* = 54) of the participants found setup C to be “very comfortable” or “comfortable” and only two participants (10%, *n* = 6) considered the comfort as “neutral”. In contrast, 8 participants (40%, *n* = 24) reported the setup R to be “uncomfortable” or “very uncomfortable”, 6 (30%, *n* = 18) found it “neutral”, and 6 (30%, *n* = 18) evaluated it as “very comfortable” or “comfortable”.

The results for pressure sensation at four positions (*n* = 240 from 60 responses for four positions) are reported in Fig. 2b. During the session, the pressure sensation increased over time; for setup C, the highest value was “light” pressure and it increased 3% (initial *n* = 25; after 30 min, *n* = 32) over time, while in setup R, “strong” pressure was the highest value, and the increase over time was of 9% (initial *n* = 3; after 30 min, *n* = 24).

With respect to sweating (Fig. 2c), there were small rises in the sensations over time. For setup C, sweating did not change between 10 min and the end of the test (after 30 min), 100% (*n* = 180, 60 responses at three time points) of the participants stated there was no sweating or it was “light”. In setup R, small changes were shown; 98.3% (*n* = 59, out of 60) described the sensation as “no” or “light”, and 1.7% (*n* = 1, out of 60) described it as “moderate” when the test finished.

In the itching evaluation (Fig. 2d), for setup C, 100% (*n* = 180, 60 responses at three time points) of the participants reported “no” or “light”, and it was stable across time. In contrast, setup R showed small rises: after 30 min of stimulation, 93.3% (*n* = 56, out of 60) of the participants reported they did not feel itching or it was “light”, and 6.7% (*n* = 4, out of 60) evaluated it as “moderate” or “strong”.

**Fig. 3:**
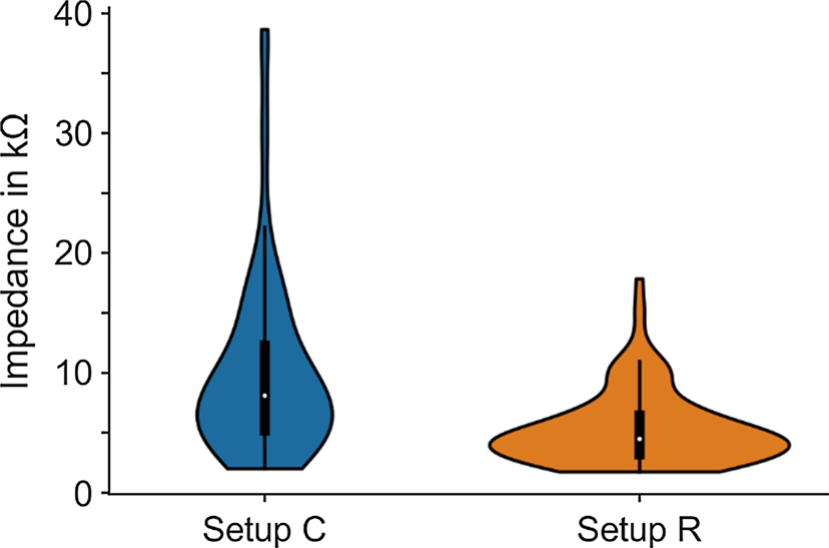
Violin plots of the impedance values derived from setup C (median 8.1 kΩ [white dot], inter-quartile range 7.1 kΩ [black box]) and setup R (median 4.5 kΩ [white dot], inter-quartile range 3.2 kΩ [black box]). Central black line indicates 1.5 times the inter-quartile range (IQR). Distributions of each *n* = 120 impedance values from two stimulation channels measured from 20 volunteers, in each three sessions

The applications of setup C and setup R were evaluated quantitatively through the use of impedance values and electrode positions. Figure 3 summarizes the results for the *n* = 120 (two stimulation channels measured from 20 volunteers, in each three sessions) impedance values derived from the sham stimulation signals, reflecting the electric contact, but not intended for treatment or intervention. In setup C, the impedance averaged 10.0 kΩ ± 4.4 kΩ (mean ± std), with a 95% confidence interval of the mean value of [8.8; 11.4] kΩ. The impedance in setup R was, on average, 5.3 kΩ ± 3.1 kΩ with a 95% confidence interval of the mean of [4.8; 5.9] kΩ.

**Fig. 4:**
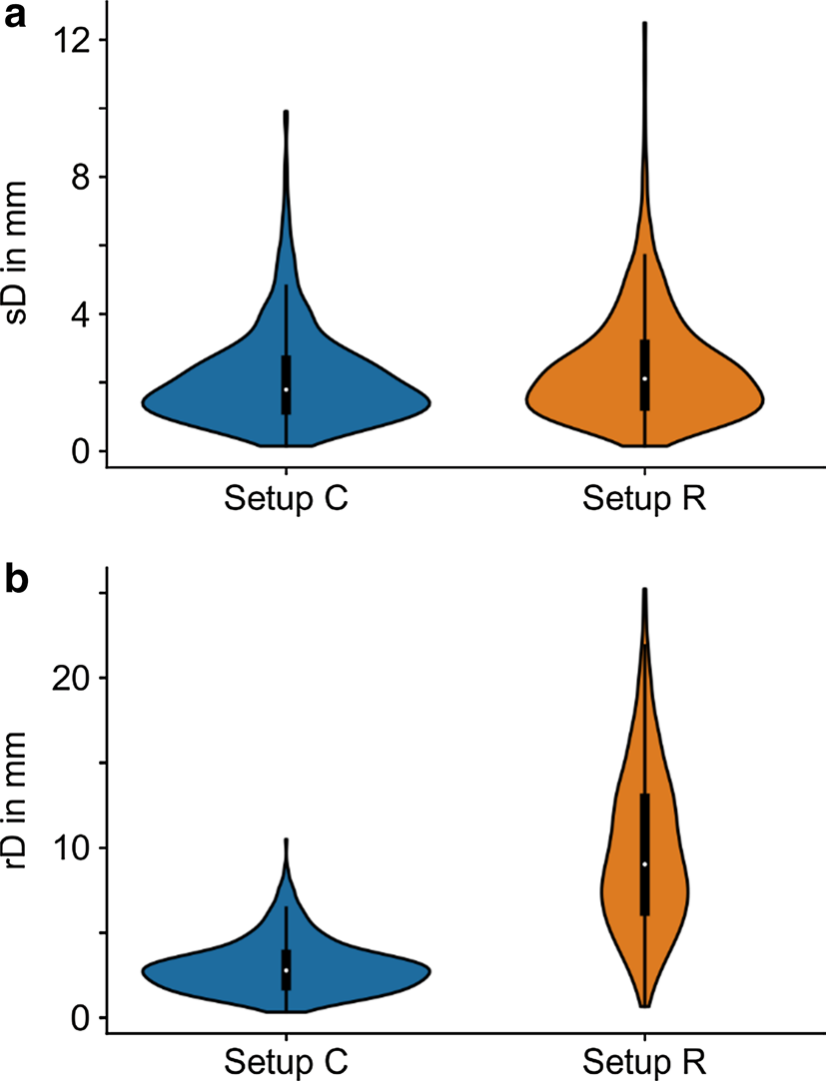
**a** Stability evaluation calculated as the Euclidean distance between electrode corner digitizations at initial position and after 30 min—setup C (median 1.8 mm [white dot], inter-quartile range 1.5 mm [black box]), setup R (median 2.1 mm [white dot], inter-quartile range 1.8 mm [black box]). Distributions of *n* = 960 Euclidean distances from 4 corners of 4 stimulation electrodes measured on 20 volunteers, each in three sessions. **b** Reproducibility evaluation calculated as the Euclidean distance between the initial electrode corner digitizations aligned across sessions—setup C (median 2.8 mm [white dot], inter-quartile range 1.8 mm [black box]) setup R (median 9.0 mm [white dot], inter-quartile range 6.6 mm [black box]). Central black lines indicate 1.5 times the inter-quartile range (IQR). Distributions of *n* = 640 Euclidean distances from four corners of four stimulation electrodes measured on 20 volunteers for two placement repetitions (session 1–session 2, session 1–session 3)

The measurements taken of the positions of the electrodes enabled the evaluation of the stability and reproducibility of the two setups. For the stability analysis (sD), the Euclidian distance between electrode positions at the start of the test and after 30 min showed a mean and standard deviation of 2.1 mm ± 1.4 mm, with a 95% confidence interval of the mean value of [2.0; 2.2] mm for setup C. Comparable values were obtained for setup R: a mean and standard deviation of 2.4 mm ± 1.6 mm and a 95% confidence interval of [2.3; 2.6] mm. Figure 4a depicts the distributions of sD with *n* = 960 Euclidean distances of four corners from four stimulation electrodes on 20 volunteers, each in three sessions.

Reproducibility (rD) was evaluated, taking into account the Euclidian distance among the initial position measurements taken in each session. Figure 4b summarizes the result for *n* = 640 Euclidean distances between positions measured in two application repetitions (session 1–session 2, session 1–session 3) from four corners of four stimulation electrodes on 20 volunteers. In setup C, the mean distance was 3.0 mm ± 1.5 mm (mean ± std), with a 95% confidence interval of the mean value of [2.9; 3.1] mm. In direct comparison, the setup R showed a significant increase, with a mean and standard deviation of 9.8 mm ± 4.7 mm and a 95% confidence interval of the mean of [9.5; 10.2] mm.

## Discussion

The newly developed flexible cap with integrated textile electrodes as an application system for tES was compared qualitatively and quantitatively to the conventional setup of rubber electrodes in sponge pockets, with respect to their usability.

The flexible knitted cap incorporated multi-compartment electrodes consisting of a contact surface of silver-coated threads, a pocket behind the fabric containing sponges soaked in saline solution, and a diffusion barrier of silicone surrounding the electrode.

**Fig. 5:**
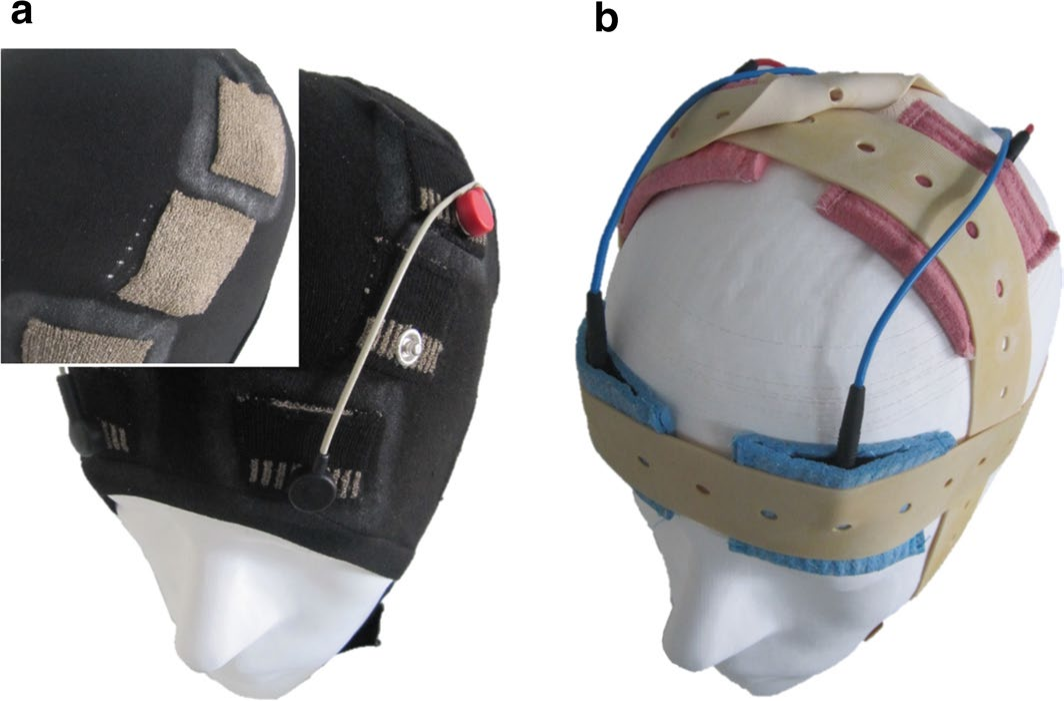
The novel flexible cap, setup C (**a**) and the electrode configurations of the conventional application system, setup R (**b**) on a head model. The inset in **a** depicts the inside of the cap with the textile electrodes exposed

Fastening conventional electrode setups by rubber bands limits the number of electrodes in use as well as the positions that can be addressed [16]. The flexible cap allows for the integration of multiple electrodes as indicated in Fig. 5. Multi-channel tES applications essential for addressing target areas [22] or administering currents from multiple sources [23] are applicable through the utilization of the flexible cap with integrated multiple electrodes. For more focal current application, electrode extent can be decreased to approximately 20 mm and electrode shapes can be adapted by angled edges.

The flexibility of the knitted fabrics allowed a very good fit to individual head shapes with an elongation of about 320% [24]. This, and the diffusion barrier infiltrated into the textile surrounding the electrode defined the desired effective electrode area, which contributes to stimulation. The textile stimulation electrodes prevented partial displacements due to bending or protrusion often perceived with conventional tES electrodes [25]. The textile electrodes integrated in the flexible cap adapted to the individual head curvatures. Thus, the electrode shape was not rigid and deformation due to uneven stretching could not be ruled out.

A simulation study [26] demonstrated the importance of electrode position and orientation for a precise tES application. The evaluation of the electrode positions showed an increase in the electrode configuration stability and reproducibility when facilitating the novel flexible cap in comparison to the conventional system [27].

The cut pattern of the cap presented in this study was optimized for a head circumference of 58 cm and an average head height, because the participants were drawn from a local student population. However, this does not limit the applicability of the cap. We are currently developing a size system considering head height and circumference to derive cap sizes for adult population and will in future also extend this to children. Moreover, the cap will be produced according to individual patient needs in lot size one, which includes customized electrode configurations and individual fitting.

The main difference of our cap to existing other cap-based tES systems is thus in the intended use. While other caps [28, 29] using gel-based Ag/AgCl electrodes aim at multiple use in many patients or volunteers, our cap will be assigned to a single person for a single medical condition. Additionally, our flexible cap with integrated textile stimulation electrodes required no skin preparation or extra electrolyte gel or paste, reducing the risk of skin irritations and hair loss [11]. Further the textile stimulation electrodes can be designed individually, largely eliminating restrictions on areas and shapes of electrodes. This can also contribute to a reduced risk of exceeding the current density limit [30] for too small electrode areas.

The aspects discussed above could improve the ease of use for patients and volunteers and potentially allow the application of the cap without trained medical personnel. The most relevant difference between the setups was in comfort and pressure grading. A fraction (14%) of the responses indicated a “modest” or “strong” pressure sensation for the conventional setup. In contrast, only one of 240 reactions indicated a “modest” feeling of pressure when the cap was applied. The participants reported that the locations for the pressure sensation were mostly associated with the rubber straps in setup R. Due to the specifics of this setup, one rubber strap crossed the forehead and the temples, which have the lowest pressure threshold on the head [31]. In setup C, the discomfort was mainly caused by the chinstrap which tightens the cap on the person’s head.

The comfort rating favored the cap, as it received 18 marks as “comfortable” or “very comfortable” and only six participants gave the same for the conventional setup. According to the participants’ responses, setup C was overall more comfortable for tES application. Further, the ratings for setup C were stable, which can help to keep the focus on potential experiments and reduce changes in the test conditions [11].

The flexible cap also allows for the integration of recording electrodes, as introduced by Wunder et al. [24]. In this study, the low preparation effort and high reproducibility were highlighted by the operators, and the participants reported a good and comfortable fit of the cap. Such advances and the achieved ease of use broaden application scenarios of the new cap to combined tES–EEG studies and home-use scenarios.

The operators’ evaluations valued the flexibility in setup C, allowing an electrode to be placed more easily that in setup R. This indicated advantages introduced by setup C, which can help to reduce training time and ease the use of tES for different operators.

In the present study, the time for fitting the electrodes onto a participant’s head showed a difference of mean values of 2.9 min in favor of setup C. In practice, setup C cut the temporal effort for trained personnel almost by a factor of two. This also decreased the time burden for participants, which is important in a clinical environment.

The stimulation signal in the present study was used to evaluate the electric contact, only. Here, no treatment or intervention was intended. Therefore, we applied the sham stimulation signal, since a stimulation at 80 Hz remained without an effect [32]. However, the influence of sham stimulation requires further investigation [33]. The impedance analysis evaluated here showed that there is a mean difference of 4.7 kΩ between both setups. The mean for setup C was 10 kΩ and for setup R was 5.3 kΩ. However, even the value of 10 kΩ is fully acceptable for a typical electric stimulation (with 2 mA), translating to a voltage of 20 V, which is within the safety limit [34]. True impedance measurements during active stimulation with the textile stimulation electrodes were introduced by Wunder et al. [24].

## Conclusion

The flexible cap with integrated textile stimulation electrodes overcomes some of the fundamental limitations of the conventional tES application systems.

## Methods

### Subjects

Twenty healthy volunteers (age: 24.0 a ± 1.6 a; 7 female) participated in the study, and two operators, without considerable prior experience in tES studies, carried out the experiment. All volunteers were asked about potential contraindications and provided written informed consent.

### Stimulation setup

The tES configuration incorporated two stimulation channels with two electrodes each. The electrodes for channel one were positioned at Fp1 and F3 and for channel two at Fp2 and F4 [35, 36].

The conventional application system, setup R, incorporated 4 cm × 4 cm rubber electrodes that were placed in saline-soaked sponges and fixated by rubber bands (Fig. 5a).

The novel flexible cap, setup C, was manufactured with a highly flexible thread of cotton and elastane. The cap was produced by flat knitting (warmX GmbH, Apolda, Germany). tES electrodes were produced with a second knitting magazine, holding conductive, silver-coated, poly-amide threads, implementing reproducible tES electrodes with respect to their size and positioning in the caps.

Pockets of the flexible fabric on top of the electrodes held sponges, sockets, and studs of snap fasteners. The latter contacted the conductive thread, which fed into the pocket’s outer side to provide an electric contact for medical-grade, press-stud cable. The saline solution used to realize the electric contact from the textile electrode to the scalp was buffered in the sponges, which provided an electrolyte reservoir. The flexible fabric surrounding the electrode with the electrolyte reservoir pocket was coated with medical-grade, low-viscosity silicone (Silpuran 2400, Wacker Chemie AG, Munich, Germany) in order to avoid a diffusion of the saline solution throughout the flexible textile. Figure 5b depicts the cap on a head model.

### Experimental procedure

Each volunteer participated in three sessions; in each, the stimulation was applied with the cap (setup C) and the conventional rubber bands (setup R) in a randomized order. The three sessions were conducted with at least a 1-day break in between. The two operators performed 30 sessions, each. The group of 20 volunteers was divided equally among the two operators, with each attending 10 volunteers.

Participants received stimulation from two channels of a DC-Stimulator MC (neuroConn GmbH, Ilmenau, Germany) for 30 min. For all stimulations, a sham protocol of a sinusoidal current with 50 μA peak to peak and a frequency of 85 Hz [15] was provided. The stimulator adjusted the driving voltage to keep the current at the desired level. Both, the applied current and the adjusted voltage were recorded with 8000 samples per second.

At the beginning and throughout the stimulation sessions (10 min, 20 min and 30 min) subjective evaluations were obtained. Participants reported their status by completing a questionnaire (Additional file 1: Appendix S1). For each application, the time to position the electrodes was obtained, and the operators evaluated the application process for each setup by completing a questionnaire (Additional file 1: Appendix S1). The four corners of each electrode were digitized with a Polaris Spectra system (Northern Digital Inc., Waterloo, Canada) immediately after application and at the end of the stimulation.

### Data processing

A FIR band-pass filter between 80 and 90 Hz, designed using a Hamming window, was applied to the recorded sinusoidal signals for the applied current and the adjusted voltage. The impedance was calculated, after an initial settling time of 10 min, using the effective current and voltage values. These effective values were obtained with signal windows of 10 sinusoidal cycles and 50% overlap. Finally, the mean impedance was calculated for each channel.

Position recordings were used for stability (sD) and reproducibility (rD) analyses. Stability was evaluated with Euclidean distances between positions within a session. Reproducibility was calculated with Euclidean distances among the three sessions of each volunteer, after aligning the positions from the second and third session to the positions from the first session, using an iterative closest-point search (Per Bergström, Matlab, The Mathworks Inc., Natick, USA).

## Supplementary information

**Additional file 1: Appendix S1.** Questionnaire: Usability evaluation of tES application systems.

## Data Availability

All data generated or analyzed during this study are included in this published article.
