# Supplementary material for: Novel flexible cap for application of transcranial electrical stimulation: a usability study
Source: Biomed Eng Online. 2020 Jun 17;19:50. doi: 10.1186/s12938-020-00792-1 (PMC7302393; doi:10.1186/s12938-020-00792-1)
Supplement: Supplementary file 1 — Additional file 1: Appendix S1. Questionnaire: Usability evaluation of tES application systems. [file 12938_2020_792_MOESM1_ESM.pdf]

Questionnaire
Usability evaluation of TES application systems

To be filled by the operator.

ID		Date			
Session		Time			
Trial		System	☐	C	☐ R

Please answer the following questions about the usability of the two application systems. Your answers will help to evaluate the stability, repeatability and manageability of the two systems.

Application of electrodes

How did you find the application of the electrodes?

☐ very comfortable ☐ comfortable ☐ neutral ☐ uncomfortable ☐ very uncomfortable

How do you evaluate the duration of the electrode application?

☐ very fast ☐ fast ☐ neutral ☐ slow ☐ very slow

Did you feel any pressure during application? If yes, where and how did you felt it.

	light	intermediate	strong
at the electrodes	☐	☐	☐
at the temples	☐	☐	☐
at the strap	☐	☐	☐
others ...	☐	☐	☐
non	☐		

During the stimulation

10 min in stimulation

Do you sweat at your head?

☐	☐	☐	☐	☐
no	slight	moderate	strong	very strong

Do you feel itching at your head?

☐	☐	☐	☐	☐
no	slight	moderate	strong	very strong

Do you feel any pressure? If yes, where and how do you feel it.

	light	intermediate	strong
at the electrodes	☐	☐	☐
at the temples	☐	☐	☐
at the strap	☐	☐	☐
others ...	☐	☐	☐
non	☐		

20 min in stimulation

Do you sweat at your head?

☐	☐	☐	☐	☐
no	slight	moderate	strong	very strong

Do you feel itching at your head?

☐	☐	☐	☐	☐
no	slight	moderate	strong	very strong

Do you feel any pressure? If yes, where and how do you feel it.

	light	intermediate	strong
at the electrodes	☐	☐	☐
at the temples	☐	☐	☐
at the strap	☐	☐	☐
others ...	☐	☐	☐
non	☐		

After the stimulation

Do you sweat at your head?

no	slight	moderate	strong	very strong
☐	☐	☐	☐	☐

Do you feel itching at your head?

no	slight	moderate	strong	very strong
☐	☐	☐	☐	☐

Do you feel any pressure? If yes, where and how do you feel it.

	light	intermediate	strong
at the electrodes	☐	☐	☐
at the temples	☐	☐	☐
at the strap	☐	☐	☐
others ...	☐	☐	☐
non	☐		

How do you evaluate the overall comfort of the application system?

very comfortable	comfortable	neutral	uncomfortable	very uncomfortable
☐	☐	☐	☐	☐
